# Mechatronic Feasibility of Minimally Invasive, Atraumatic Cochleostomy

**DOI:** 10.1155/2014/181624

**Published:** 2014-07-07

**Authors:** Tom Williamson, Xinli Du, Brett Bell, Chris Coulson, Marco Caversaccio, David Proops, Peter Brett, Stefan Weber

**Affiliations:** ^1^ARTORG Center for Biomedical Engineering Research, University of Bern, 3010 Bern, Switzerland; ^2^Brunel Institute for Bioengineering, Brunel University, Uxbridge UB8 3PH, UK; ^3^Department of Otolaryngology, Queen Elizabeth Hospital, Birmingham B15 2TH, UK; ^4^Department of ENT, Head and Neck Surgery, Inselspital, University of Bern, 3010 Bern, Switzerland

## Abstract

Robotic assistance in the context of lateral skull base surgery, particularly during cochlear implantation procedures, has been the subject of considerable research over the last decade. The use of robotics during these procedures has the potential to provide significant benefits to the patient by reducing invasiveness when gaining access to the cochlea, as well as reducing intracochlear trauma when performing a cochleostomy. Presented herein is preliminary work on the combination of two robotic systems for reducing invasiveness and trauma in cochlear implantation procedures. A robotic system for minimally invasive inner ear access was combined with a smart drilling tool for robust and safe cochleostomy; evaluation was completed on a single human cadaver specimen. Access to the middle ear was successfully achieved through the facial recess without damage to surrounding anatomical structures; cochleostomy was completed at the planned position with the endosteum remaining intact after drilling as confirmed by microscope evaluation.

## 1. Introduction

Surgery on the lateral skull base provides an ideal environment for the integration of surgical robotics and computer assisted surgical techniques due to the rigidity of anatomical structures and the desire for high precision and accuracy. Recent technological advances have led to an increasing amount of research in this field; cochlear implantation procedures in particular have been a topic of great interest for the integration of robotic assistance. Cochlear implantation, in which an electrode is inserted into the cochlea in order to directly stimulate the auditory nerve, can be a time-consuming and demanding procedure. The surgeon first mills away a large portion of the mastoid region of the temporal bone before reaching the facial recess, a region bordered by the facial nerve, external auditory canal, and chorda tympani; each of these structures is then visually identified. Final access to the middle ear is then obtained by drilling through the space between these structures, a space typically only 2-3 mm wide. The surgeon must then insert the electrode through this cavity into the cochlea through a window either natural (the round window) or artificial (cochleostomy).

Robotic assistance has been proposed for various elements of the cochlear implantation procedure, including systems designed to minimize the invasiveness of access to the middle ear or to help preserve residual hearing by ensuring the integrity of intracochlear structures and minimizing intracochlear trauma. The former systems typically attempt to minimize the size of the mastoidectomy or remove this step entirely by replacing it with a single tunnel drilled from the surface of the mastoid to the inner ear through the facial recess, so-called direct cochlear access (DCA) or percutaneous cochlear implantation (PCI). A number of approaches have been evaluated including patient specific templates [1, 2], modified industrial robots [3], and custom robotic systems, in both head-mounted parallel [4, 5] and bed-mounted serial manipulator configurations. Initial investigation related to DCA focused largely on the accuracy required to safely complete the procedure, which has proven prohibitively high due to the size of the facial recess. The patient specific template approach has demonstrated sufficient accuracy and is currently undergoing clinical evaluation [6]; more recently a custom robotic system also demonstrated the necessary level of accuracy in an in vitro cadaver trial [7].

The preservation of residual hearing in patients undergoing cochlear implantation has also been the subject of significant research in the recent past. Clinical considerations such as the ideal route for implantation, whether through the round window (minimizing initial trauma) or a cochleostomy (allowing insertion along trajectories tangent to the basal turn of the cochlea), are currently the subject of extensive debate with no consensus yet reached [8–11]. The formation of the cochleostomy is thought to be one of the key components in preserving hearing [12]. Lenhardt recommends that a bony cochleostomy be created with a slow turning burr. The underlying endosteal membrane is preserved and opened with a knife/pick rather than the running burr to reduce the trauma to the cochlea and prevent contamination of the scala tympani with blood and bone dust. In parallel with the debate surrounding these clinical factors, a number of groups have been working towards minimizing intracochlear trauma through the use of robotics. Trauma minimization through the use of insertion tools, which typically involve the measurement and minimization of applied forces during insertion, has been extensively described [13–15]. The reduction of initial trauma due to the creation of a cochleostomy or extension of the round window through the use of robotics has also been a subject of interest.

This work describes the preliminary work on the combination of a high accuracy robotic system, designed specifically for minimally invasive access to the cochlea through a facial recess approach, and a robotic “smart drilling” system designed for the completion of a safe, minimally invasive cochleostomy. Methodologies for combination of the systems are considered and validated, and the potential for and implications of a minimally invasive, atraumatic approach to cochlear implantation are discussed.

## 2. Materials and Methods

### 2.1. Robotic System for Minimally Invasive Cochlear Access

A custom robotic system designed specifically for minimally invasive cochlear implantation and simple integration into existing operating room (OR) environments was utilized; hardware components include a lightweight (5.5 kg) robotic arm, mounted directly to the side rails of a standard OR table along with a noninvasive head holding system, which utilizes inflatable pads to fix the head of the patient relative to the robot base. A commercially available high accuracy tracking system (CamBar B1, Axios3d, Germany) is combined with custom active tracking markers for visual servoing control of the robotic manipulator. Stand-alone, custom planning software allows the visualization of preoperative imaging data and guides the surgeon through the steps required to extract the positions of implanted fiducial screws, segment vital anatomical structures, and plan a safe trajectory. Custom navigation software guides the surgeon through the steps required to successfully complete the minimally invasive procedure.

The accuracy of the robotic system was previously demonstrated in an in vitro study on 10 human temporal bone specimens; an end-to-end system accuracy of 0.15 ± 0.08 mm at a target on the cochlea was observed [7]. Additionally, the system includes a number of integrated safety features to ensure the integrity of the anatomical structures of the facial recess. Force-based pose estimation is a novel method of estimating the position of the drilling tool within the mastoid in real time during drilling. Based on the correlation of observed drilling forces with the heterogeneous nature of the mastoid, the algorithm has demonstrated the ability to estimate the position of the tool with an accuracy of 0.29 ± 0.14 mm at the level of the facial nerve [16]. Facial nerve monitoring, a safety feature commonly used during conventional cochlear implantation procedures, is also integrated into the robotic system. Initial animal trials, utilizing sheep as a model, revealed that the system as it currently exists does not have the specificity or sensitivity required [17]; however improvement is currently underway.

### 2.2. Smart Drilling System for Minimally Invasive Cochleostomy

**Figure 1:**
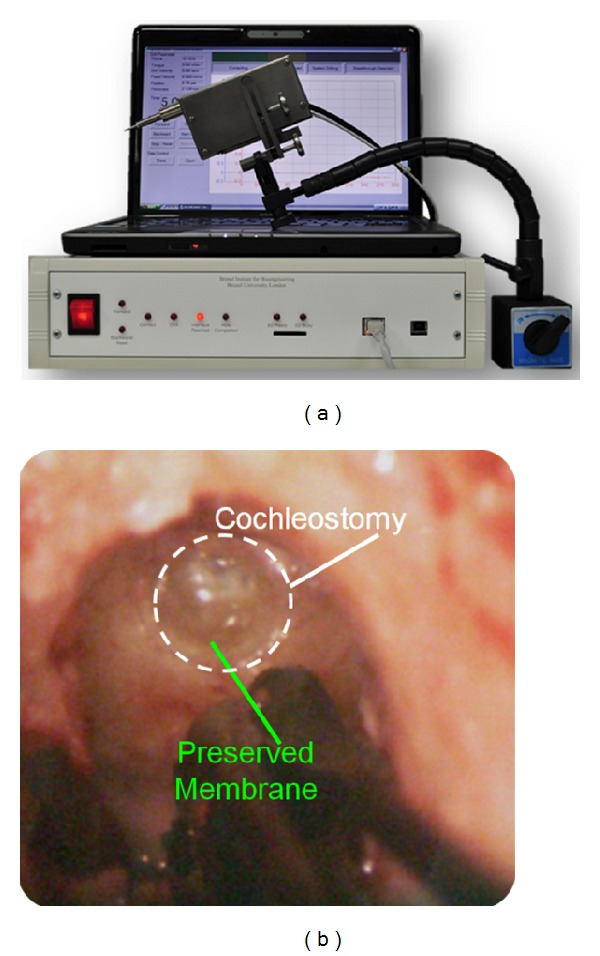
The surgical smart drilling system is capable of drilling an atraumatic cochleostomy, allowing access to the inner ear for cochlear electrode array insertion.

**Figure 2:**
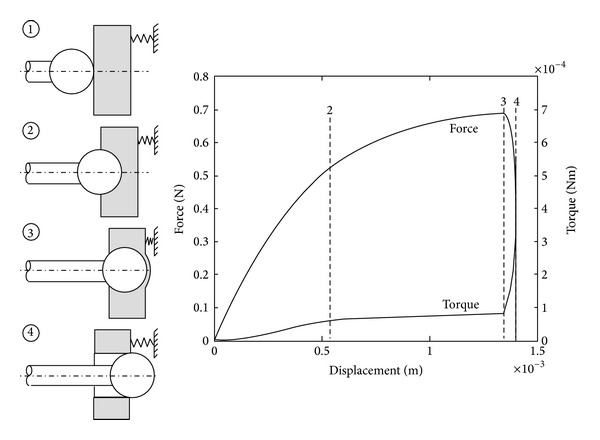
Simulated force and torque measurements during the drilling of a cochleostomy. The process can be separated into engagement, drilling, and partial and complete breakthrough stages.

The surgical smart drill system is the mechatronic system used to produce consistent windows onto the endosteum. This system enables manual positioning onto the drilling trajectory and then the automatic feed control of the tool point with respect to the deforming tissue and avoiding penetration through the endosteal membrane, as shown in Figure 1. The control strategy of the smart drill is based on the discrimination of simultaneous features in the force and torque transients during drilling. The onset of breakthrough causes a sharp result in the increase of torque signal and simultaneous roll-off of the feed force signal (shown as stage 3 in Figure 2 [18, 19]). While these simultaneous force transient features are always present when approaching a tissue interface, the values and prominence of the peaks in force and torque vary according to stiffness, drill feed velocity, tissue hardness, and sharpness of the drill bit. The control algorithms use the simultaneous features to discriminate the drilling conditions, tissues, and tissue interfaces to enable precision in the process. The robotic system integrates the drilling machine that accommodates actuators for feed and drill rotation, sensing methods for force and torque, standard drill bits, an adjustable support arm, a remote pendant, an electronics box accommodating the main processor, sensory discrimination algorithms, control strategy selection, amplifiers, and indicators showing the state of the process. A laptop can be connected to provide further information.

Preserving an intact endosteum during cochleostomy formation has the considerable advantage of reducing trauma within the cochlea. It is also possible to remove debris and insert antiseptic gel to achieve sterile conditions during the insertion of an electrode. It has also recently been shown that, in contrast with the amplitude of pressure disturbances induced in the cochlea when using a conventional surgical smart drill, the amplitude of disturbances induced within the cochlea is only 1% of that when drilling using the robotic surgical microdrill [20, 21].

## 3. Experimental Protocol

### 3.1. Preoperative Imaging and Planning

One human whole head cadaver specimen was prepared bilaterally with four fiducial screws. Preoperative cone beam CT (CBCT) images were then obtained with the screws in place (ProMax, PlanMeca Finland, voxel size 0.15 mm isometric) and trajectories were planned through the facial recess on both the left and right sides using custom planning software [22]. The target point was defined within the middle ear cavity, with the trajectory directed to a position on the cochlea as per instructions from an experienced otologic surgeon; the cochleostomy position was confirmed by the same surgeon.

### 3.2. Direct Cochlear Access

The specimen was fixed to a standard OR table (Schaerer Switzerland) using a noninvasive head holding device, with the robot mounted beside it. A reference marker was rigidly attached to the patient and registration was performed as described previously [23].

The robot then followed the planned trajectory under the supervision of the user and drilled a DCA tunnel to the middle ear space, stopping just before contact was made with the promontory (feed rate 0.5 mm/sec and rotational speed 5000 RPM). Once access to the middle ear was obtained, the robot was removed from the surgical site, and the patient reference marker was removed.

### 3.3. Cochleostomy

The specimen was removed from the head clamp and placed on a surgical workstation with microscope. The tympanic membrane was removed to allow visualization of the cochleostomy target site, and the DCA tunnel was cleared of debris using a combination of water injection and suction. Next, a standard 1 mm diameter diamond burr was attached to the smart drilling system, where after the drill was manually aligned with the previously drilled DCA tunnel and inserted to a depth of 1 mm above the surface of the cochlea and held in place with the adjustable arm, small adjustments were made to the position of the burr to ensure that no contact between the burr shaft and the DCA tunnel was present. The cochleostomy was then autonomously drilled by the smart drill system. Drilling was completed at a base feed rate of 0.008 mm/s and rotational speed of 9000 RPM. A maximum drilling force of 2.5 N was defined, with the feed rate automatically altered to ensure forces did not exceed this value. Following the completion of the automated drilling, the surgeon confirmed the integrity of the endosteum through visual inspection once the drill was removed.

**Figure 3:**
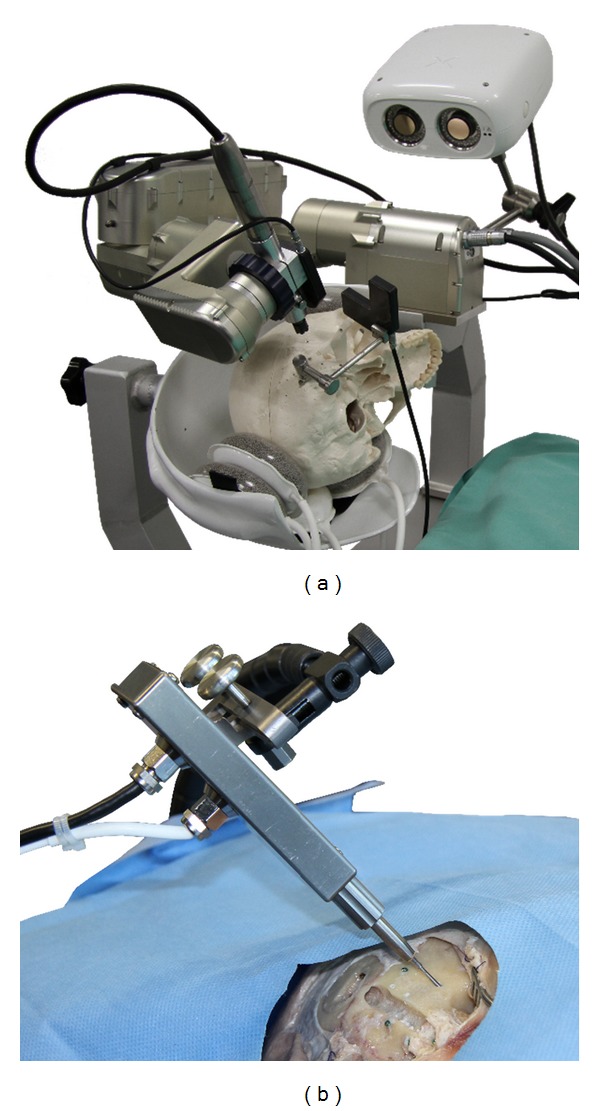
The robotic system for minimally invasive cochlear access (a) and smart drilling tool for cochleostomy, inserted through a drilled DCA trajectory (b).

The setup for both intraoperative components of the procedure is shown in Figure 3.

## 4. Results

**Figure 4:**
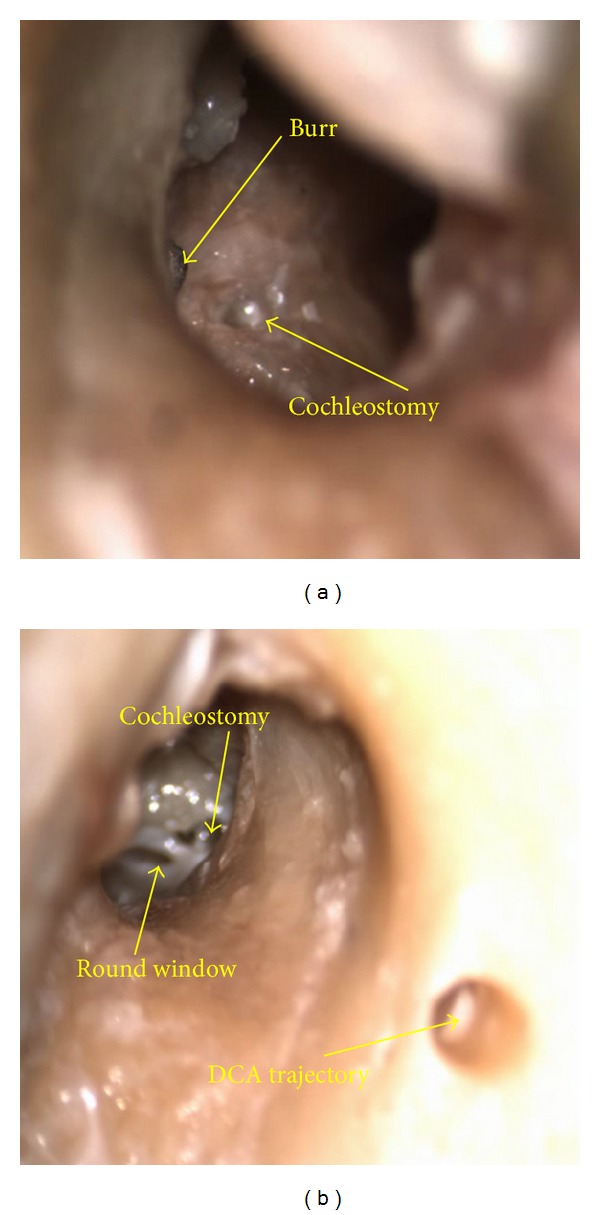
Microscope views through the external auditory canal of cochleostomy (a) and extended round window (b) completed through drilled DCA trajectories (visible in the right image); the burr is visible in the left image.

Access to the inner ear was successfully achieved in both cases. The endosteum remained intact after the completion of the cochleostomy in both cases as confirmed intraoperatively by an experienced surgeon through microscopic inspection as shown in Figure 4.

It was determined after completion of the minimally invasive access and subsequent insertion of the cochleostomy drill through the tunnel that the selected cochleostomy sites were not ideally located in both cases. In the first case (Figure 4(b)), the cochleostomy site was located too anterior and too far along the basal turn of the cochlea. In the second case (Figure 4(a)), the target was located too posterior and too close to the round window niche; however it was still possible to drill a round window extension.

## 5. Discussion

Debate regarding the best route for cochlear insertion, whether directly through the round window or after the creation of a cochleostomy, is ongoing. While the round window approach has the advantage of avoiding immediate trauma to the inner ear as no drilling is required, the observed angle of insertion relative to the axis of the basal turn of the cochlea may not be ideal, potentially leading to penetration of the basilar membrane or insertion into the scala vestibuli. Manual cochleostomy is by its very nature more traumatic than a round window approach; excess penetration into the cochlea may lead to structural damage. Adunka et al. [24] demonstrated that in many cases the drilling of a cochleostomy directly damages the basal structures, as well as the addition of foreign particles such as bone fragments into the cochlea. However, an accurately placed cochleostomy may provide a better insertion angle compared to a round window approach, as shown in [25], subsequently reducing the likelihood of trauma later on in the insertion process. Berrettini et al. [26] demonstrated that the drilling of a modified anterior inferior cochleostomy combined with the use of an Advance Off-Stylet type electrode allowed retention of residual hearing in 81.8% of patients (*n* = 11), as compared to only 25% through a round window approach (*n* = 10). The smart drilling tool presented within allows the placement of a cochleostomy without many of the problems associated with the manual drilling of the cochlea. By keeping the endosteum intact, excess penetration and damage are avoided and the sterility of the inner ear is maintained. A recent study has also shown that robotic drilling leads to significantly decreased disturbance levels (as measured by laser Doppler vibrometry) when compared to manual drilling, potentially preserving residual hearing.

The combination of the smart drill with the robotic system could enable a more fully integrated minimally invasive procedure spanning minimally invasive access through the facial recess and atraumatic cochleostomy. The planning software, which allows the segmentation of vital anatomical structures from preoperative imaging data and the selection of a drilling trajectory, is currently optimized for a round window approach; in order to define the target position the user is instructed to select the center of the round window. Insertion of electrodes through a minimally invasive round window approach, without utilizing specialized insertion tools, has been shown to be feasible [27]. However the completion of a cochleostomy introduces a number of additional considerations for trajectory definition, particularly regarding the optimal site for the cochleostomy and the relative angle between the trajectory and the basal turn of the cochlea. Modifications to the planning software which simplify the selection of the site and trajectory orientation are the topic of ongoing work.

The smart drilling tool has proven to be robust to a variety of differences in operating environment; the endosteum is preserved and cochleostomy completed even when drilling at angles of up to 45° relative to the surface of the cochlea, as well as when the system is knocked or disturbed during drilling [19]. The angular robustness in particular is of vital importance as the DCA tunnel significantly constrains the possible cochleostomy drilling angle. While some skating of the drill was noted in the first case, the cochleostomy was still successfully completed without penetration of the endosteum. The exact effects of fixation on the physiological characteristics of the temporal bone specimens are unknown; however the smart drill system for cochleostomy has previously been tested on patients demonstrating that it can effectively preserve the endosteal membrane through a standard mastoidectomy [20]. The robotic system for minimally invasive cochlear access has previously demonstrated sufficient accuracy for the safe completion of drilling through the facial recess. Preparation for a pilot trial utilizing the system is currently underway.

Ongoing work includes further improvement of system integration; insertion of the drill through the tunnel proved challenging and required the modification of standard surgical burrs due to the excessive diameter of the shaft with respect to the DCA tunnel. Friction between the shaft of the drill and the sides of the tunnel increased the torque observed by the smart drill, preventing the drill from starting; subsequently, it was necessary to align the drill almost exactly along the center of the drilled tunnel. These issues added significant time to the procedure; the intraoperative portion of the minimally invasive access takes approximately 20 minutes to complete, including system setup, registration, and drilling [7], as well as an additional 15 minutes preoperatively for anatomical segmentation and planning of the trajectory. The problems encountered during the drilling of the cochleostomy added an additional 20–30 minutes to the intraoperative time; it is expected that this can be reduced significantly through further integration of the two systems.

Consolidation of the operating principles of the two systems may be possible; the DCA robotic system includes a six-axis force-torque sensor at the wrist, currently used for the measurement of drilling forces for the force-based pose estimation safety feature. The algorithms utilized by the smart drilling system may theoretically be applied to the forces and torques observed by the robotic system; however significant validation is required before clinical use would be possible.

## 6. Conclusions

This work has presented preliminary work on the combination of two robotic systems for reducing invasiveness and trauma in cochlear implantation procedures. A robotic system for minimally invasive inner ear access was combined with a smart drilling tool for robust and safe cochleostomy, and evaluation was completed on a single human cadaver specimen. Access to the middle ear was successfully achieved through the facial recess without damage to surrounding anatomical structures; cochleostomy was completed at the planned position with the endosteum remaining intact after drilling as confirmed by microscope evaluation.
